# Nevus Lipomatosus Superficialis on the Left Proximal Arm

**DOI:** 10.1155/2017/6908750

**Published:** 2017-09-13

**Authors:** Alexander K. C. Leung, Benjamin Barankin

**Affiliations:** ^1^Department of Pediatrics, The University of Calgary, Calgary, AB, Canada T2M 0H5; ^2^The Alberta Children's Hospital, Calgary, AB, Canada T2M 0H5; ^3^Toronto Dermatology Centre, Toronto, ON, Canada M3H 5Y8

## Abstract

We report a 58-year-old woman with a solitary type of nevus lipomatosus superficialis on the left proximal arm. To our knowledge, the occurrence of a solitary type of nevus lipomatosus superficialis on the arm has very rarely been reported. A perusal of the literature revealed but one case, to which we are going to add another one. Recognition of this clinical manifestation is important so that a proper diagnosis can be made.

## 1. Introduction

Nevus lipomatosus superficialis is a hamartoma characterized by ectopic mature adipose tissue in the papillary dermis. The condition was first described by Hoffman and Zurhelle in 1921 [1]. Two types of nevus lipomatosus cutaneous superficialis are recognized, namely, the classical multiple type (also known as the Hoffman-Zurhelle type) and the solitary type [2, 3]. We describe a 58-year-old woman with a solitary type of nevus lipomatosus superficialis on the left proximal arm. To our knowledge, the occurrence of a solitary type of nevus lipomatosus superficialis on the arm has very rarely been reported.

## 2. Case Report

A 58-year-old woman presented with a slow-growing multilobulated plaque on the left proximal arm of approximately 2 years' duration. The lesion was asymptomatic with no history of ulceration or discharge. There was no history of preceding trauma. The patient was otherwise in good health. Family history was noncontributory.

**Figure 1:**
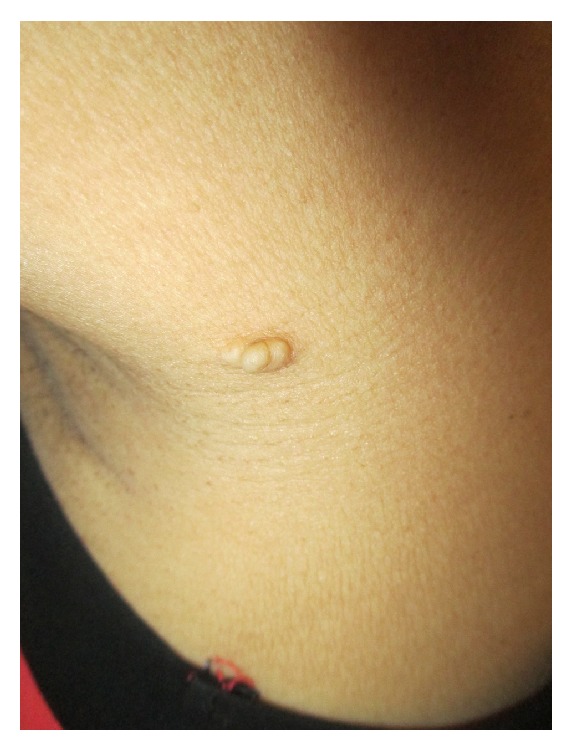
A soft, skin-colored, multilobulated, well-defined, discrete, dome-shaped plaque on the left lateral proximal arm adjacent to the axilla.

Physical examination revealed a soft, skin-colored, multilobulated, nontender, well-defined, discrete, dome-shaped plaque on the left lateral proximal arm adjacent to the axilla (Figure 1). There was no axillary lymphadenopathy. There was no comedo-like lesions, café au lait macules, or hypertrichosis on the surface of the lesion. The rest of the physical examination was normal.

**Figure 2:**
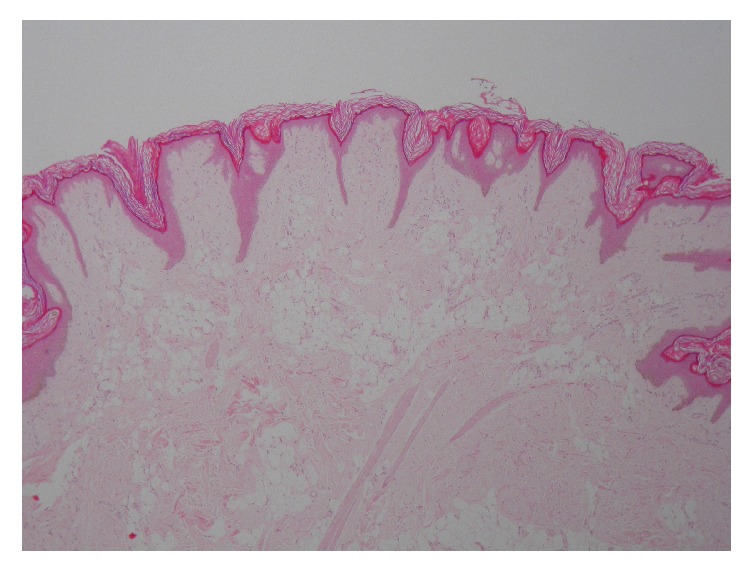
Histopathology showing islands of mature adipocytes in the papillary dermis.

Excisional biopsy of the lesion showed islands of mature adipocytes in the papillary dermis, confirming the clinical diagnosis of nevus lipomatosus superficialis (Figure 2).

## 3. Discussion

Nevus lipomatosus superficialis is an uncommon condition. In a retrospective study, 8 cases were seen in an 11-year-period from 2001 to 2011 at the Department of Dermatology, Venerology and Leprology, Postgraduate Institute of Medical Education and Research in Chandigarh, India [4]. In another retrospective study, 8 cases were seen in a 14-year-period from 1997 to 2010 at the Department of Dermatology, Charles Nicole Hospital in Tunis, Tunisia [5]. There is no familial or sex predilection [3].

The condition is usually idiopathic and the exact pathogenesis is not known [2]. Theories such as mesenchymal perivascular differentiation of lipoblasts, focal heterotopic development of adipose tissue, and adipose metaplasia in the course of degenerative changes of dermal collagen bundles and elastic tissue have been proposed to account for the heterotopic occurrence of adipose tissues but not substantiated [4, 5]. Deletion of 2p24 has been described, suggesting that genes may have a role to play [6].

The classical type is usually present at birth or appears within the first two decades of life [3]. Typically, the classical type presents as asymptomatic, multiple, soft, yellowish or skin-colored, sessile or pedunculated, papules or nodules, often coalescing into plaques whose surface may be smooth, wrinkled, or cerebriform or have a peau d'orange appearance that are present in a linear, zosteriform, or segmental pattern [3, 7]. Sites of predilection include the pelvic girdle, gluteal region, lower trunk, and upper thigh [3, 7].

The solitary type, on the other hand, often appears between the third and sixth decade of life and typically presents as an asymptomatic, solitary, yellowish to skin-colored, dome-shaped or sessile papule or nodule, as is illustrated in the present case [7]. The lesion has no predilection for any particular site and can develop anywhere on the body, including the scalp, neck, face, eyelid, nose, knee, axilla, arm, scrotum, vulva, and clitoris [3, 7–11]. The occurrence on the arm has been rarely reported and a perusal of the literature revealed only one case. In 1975, Jones et al. reported a male patient with a solitary nevus lipomatosus superficialis on the arm [11]. The authors, however, did not specify the age of the patient and the laterality of the lesion. The occurrence of the lesion on the arm may be more common than is presently appreciated. Recognition of this clinical manifestation is important so that a proper diagnosis can be made.

## 4. Conclusion

Although the solitary type of nevus can develop anywhere on the body, the occurrence on the arm has rarely been reported; a perusal of the literature revealed only one case. The occurrence of the lesion on the arm may be more common than is presently appreciated. Recognition of this clinical manifestation is important so that a proper diagnosis can be made.
